# Transcriptome Profiling to Understand the Effect of Citrus Rootstocks on the Growth of ‘Shatangju’ Mandarin

**DOI:** 10.1371/journal.pone.0169897

**Published:** 2017-01-12

**Authors:** Xiang-Yu Liu, Juan Li, Meng-Meng Liu, Qing Yao, Jie-Zhong Chen

**Affiliations:** 1 College of Horticulture, South China Agricultural University, Guangzhou, Guangdong, China; 2 Qingdao Agricultural University, Qingdao, Shandong, China; 3 Department of Horticulture, Zhongkai University of Agriculture and Engineering, Guangzhou, Guangdong, China; Universidade de Lisboa Instituto Superior de Agronomia, PORTUGAL

## Abstract

To obtain insight into potential mechanisms underlying the influence of rootstock on scion growth, we performed a comparative analysis of ‘Shatangju’ mandarin grafted onto 5 rootstocks: Fragrant orange (*Citrus junons* Sieb. ex. Tanaka), Red tangerine (*Citrus reticulata* Blanco), ‘Shatangju’ mandarin (*Citrus reticulata* Blanco), Rough lemon (*Citrus jambhiri* Lush) and Canton lemon (*Citrus limonia* Osbeck). The tree size of ‘Shatangju’ mandarin grafted onto Canton lemon and Rough lemon were the largest, followed by self-rooted rootstock trees, and the lowest tree sizes correspond to ones grafted on Red tangerine and Fragrant orange rootstocks. The levels of indoleacetic acid (IAA) and gibberellin (GA) were significantly and positively related to growth vigor. The differences of gene expression in leaves of trees grafted onto Red tangerine, Canton lemon and ‘Shatangju’ mandarin were analyzed by RNA-Seq. Results showed that more differentially expressed genes involved in oxidoreductase function, hormonal signal transduction and the glycolytic pathway were enriched in ‘Red tangerine vs Canton lemon’. qRT-PCR analysis showed that expression levels of *ARF1*, *ARF8*, *GH3* and *IAA4* were negatively correlated with the growth vigor and IAA content. The metabolism of GA was influenced by the differential expression of *KO1* and *GA2OX1* in grafted trees. In addition, most of antioxidant enzyme genes were up-regulated in leaves of trees grafted onto Red tangerine, resulting in a higher peroxidase activity. We concluded that different rootstocks significantly affected the expression of genes involved in auxin signal transduction pathway and GA biosynthesis pathway in the grafted plants, and then regulated the hormone levels and their signal pathways.

## Introduction

Citrus is now grown in more than 140 countries in tropical, subtropical and Mediterranean region, and the total output of citrus in the world was at over 135 million tons in 2013 (the FAO) [1]. Being the basis of the grafted fruit trees, the rootstock has a significant impact on horticultural and pathological traits of citrus cultivars. The effect of rootstocks is particularly significant on tree nutrition [2], growth vigor [3], stress resistance [4], and fruit quality [5] of the scion. The rootstock use thus holds an important position in fruit cultivation. The study of rootstocks has been a major topic in citrus research for nearly half a century and has rapidly been developed worldwide [6]. The rational use of rootstocks can enhance the stress resistance, regulate the production period, and improve the fruit quality of citrus. Therefore, systematic and thorough studies on citrus rootstocks are important to the citrus industry development.

The growth and development of plants as well as their morphology are under strict control of relevant genes. Rootstocks can modulate the growth of grafted trees by affecting the gene expression patterns in the scion [7, 8]. Li et al. [9] found that the expression of the polar auxin transport-related gene, *PIN1*, was markedly reduced in shoots of apple grafted onto a dwarf rootstock; the change in gene expression decreased the polar transport of the indole-3-acetic acid (IAA) from the top down and thus resulted in an inadequate supply of IAA to the roots of the apple. Jensen and colleagues used cDNA Amplified Fragment Length Polymorphism (AFLP) [10] and gene chip technology [8] to investigate gene expression in shoot apices of ‘Gala’ apple grafted onto different rootstocks; they identified a number of differentially expressed genes (DEGs, e.g., *ZIP* and *EXP*) that are associated with the growth vigor of the plants. Moreover, studies have examined gene-expression differences in various scion–rootstock combinations of sweet cherry [7] and grape [11].

The transcriptome is the combined set of all transcripts expressed by an organism, and transcriptomics is the study of gene expression at the RNA level [12]. Transcriptome sequencing technologies, which lay a foundation for transcriptomic studies, mainly include gene chip technology, serial analysis of gene expression, massive parallel sequencing, and the latest high-throughput RNA sequencing (RNA-Seq) [13]. Owing to its high-throughput and low cost, RNA-Seq based on deep sequencing has been extensively used in biology, medicine, agronomy, and various other fields [14–16]. Presently, transcriptomics is being gradually applied to the study of the molecular biology of citrus, including the mechanisms of fruit color changes [17], fruit development [18], flowering [19], and disease resistance [20, 21].

Research has investigated the effect of citrus rootstocks on the scion mainly by focusing on the nutritional and hormonal regulation related to physiological characteristics. Little effort has been dedicated to study the effects of citrus rootstocks at the molecular level. In the present study, we used RNA-Seq to evaluate the effects of different rootstocks on gene expression in the leaves of the ‘Shatangju’ mandarin (*Citrus reticulata* Blanco). We analyzed the gene regulatory networks and identified the key genes through which the rootstocks induce growth differences in the scion. This study contributes to the molecular understanding of the mechanism through which rootstocks affect the scion growth of ‘Shatangju’ mandarin.

## Materials and Methods

### Plant material and experiment procedures

The experiment was conducted in Guangzhou, China (Latitude 23°16′ N; longitude 113°36′ E) in 2012–2013. The rootstock cultivars included the following: Fragrant orange (*Citrus junons* Sieb. ex. Tanaka), Red tangerine (*Citrus reticulata* Blanco cv. Red tangerine), Rough lemon (*Citrus jambhiri* Lush), Canton lemon (*Citrus limonia* Osbeck), and ‘Shatangju’ mandarin (*Citrus reticulata* Blanco).

Rootstock seeds from self-pollination were provided by the Citrus Research Institute of the Chinese Academy of Agricultural Sciences (Chongqing, China). After being germinated, uniform seeds were sown in a nursery box at the end of January 2012. Seedlings with uniform growth were chosen from each rootstock cultivar in early April 2012 and transplanted into wooden planting boxes (length × width × height, 200 × 60 × 50 cm) in network room. Three boxes were planted for each rootstock cultivar, with 15 plants per box. The experimental soil was prepared uniformly (garden soil: peat: sand = 3:2:1) and under conventional management. Virus-free budwood of ‘Shatangju’ mandarin was grafted onto these rootstocks in January 31, 2013. The grafted ‘Shatangju’ mandarin plants were subjected to conventional management in network room.

### Determination of growth parameters of grafted plants

From April 2013, shoot length (from graft union point to scion shoot apex) was measured for each rootstock treatment every month. On November2013, 10 grafted saplings were then chosen from each rootstock treatment, and the whole plants were gently removed from the planting box with soil (to avoid roots damage). The soil was carefully removed from the roots with water. The weights of the aboveground part and roots were measured. A vernier caliper was used to measure scion and rootstock trunk diameters as well as the internodal length. The rootstock–scion diameter ratio and root–shoot ratio were calculated. The area of a single leaf was measured using the LI-3100C Area Meter. Samples were collected from four parts: leaves (at the fourth to fifth node from the shoot apex), scion bark (above the graft union point), rootstock bark (between the graft union point and the main root), and roots.

### Phytohormone determinations

Phytohormones were extracted from the leaf, scion bark, rootstock bark, and root samples of ‘Shatangju’ mandarin grafted onto the different rootstocks with three biological replicates. 500 mg of fresh tissues were grinded into homogenates in liquid nitrogen, extracted with 80% (v/v) methanol for 4 h at 4°C in the darkness, and then, centrifuged for 15 min at 6000 rpm before the supernatants were collected. The residue was extracted a second time as above, and the supernatants were combined. The supernatants were purified on a C18 Sep-Pak cartridge (Waters, Milford, MA, USA), evaporated to dryness in vacuum, and redissolved in a phosphate-buffered saline solution (pH 7.5). The determination of endogenous levels of indole-3-acetic acid (IAA), gibberellin (GA), zeatin riboside (ZR) and abscisic acid (ABA), by an enzyme-linked immunosorbent assay (ELISA) technique were performed as previous publications [22]. The absorbance of each well was measured at 490 nm using a Microplate Reader (Infinite M200, Tecan, Austria). Two replications were performed for each measurement.

### RNA preparation for mRNA-Seq

Leaves (at the fourth to fifth nodes from the apices of the mature autumn shoots) were collected from ‘Shatangju’ mandarin grafted onto Red tangerine, ‘Shatangju’ mandarin, and Canton lemon. Total RNA was extracted from leaf samples with the Trizol Reagent, according to the manufacturer's protocol (TaKaRa, Japan). Before performing mRNA-Seq library construction and RNA sequencing, the quality of the RNA extracts was checked by Agilent Bioanalyzer 2100 (Agilent technologies Santa Clara, US), NanoDrop ND-1000 spectrophotometry and electrophoresis.

### Library construction and sequencing

The cDNA library construction and sequencing were performed according to Jiang et al [23]. Oligo(dT) magnetic beads were used to enrich mRNA in the total RNA extracts of the leaf samples. The mRNA was fragmented by adding a fragmentation buffer and then served as a template for cDNA synthesis. The products were purified using the QIAquick PCR Kit and eluted with EB buffer, followed by end repair, addition of a poly(A) tail, and ligation of an adaptor sequence. The final products were subjected to agarose gel electrophoresis, and the target fragments were recovered for PCR amplification. The constructed library was sequenced using the Illumina HiSeq™ 2500 System by Shanghai Biotechnology Corporation (Shanghai, China).

### mRNA-Seq data analysis

Tens of thousands of raw reads were retrieved by sequencing the constructed library. High-quality clean reads without noise were obtained by the removal of data including the adaptor sequence, sequences containing unknown bases (N) of greater than 10%, and low quality sequences.

After preprocessing, the TopHat Spliced mapping algorithm was used for mapping reads [24]. The algorithm allows segmentation and partial mapping of reads that could not be matched for the full length. This algorithm is therefore suited for the analysis of sequencing data of the eukaryotic transcriptome. Here, sequence alignment allowed two mismatches, and each read allowed multi hits ≤1. The genome version was JGI_C.clementina_v1.0 and the species was Clementine (*Citrus reticulata* Blanco). Gene coverage and sequencing saturation were analyzed to assess the quality of the sequencing.

Cufflinks was used to quantify gene expression based on the match results of TopHat [25]. The procedure was as follows: first, the specific location of the gene was obtained from the existing gene annotation file; next, the reads being covered in the gene region were counted; and finally, the FPKM (fragments per kilobase of exon model per million mapped reads) algorithm was used for standardized calculation of the gene-expression levels. The FPKM method can eliminate the effect of gene length and sequencing amount on the calculation of gene expression. The calculated gene expression levels thus can be used for directly comparing gene expression between the different samples.

The DEGseq package was used for DEG (Differentially Expressed Gene) analysis among the samples based on the normalized FPKM values. The degree of difference in DEGs was screened by fold change and Fisher’s exact test. We used a FDR (false discovery rate) < 0.05 and an absolute value of the log_2_ ratio >1 as the threshold to determine the significant difference in gene expression. Based on the nonredundant (NR) annotations, Blast2GO was used to assign unigenes to gene ontology (GO) terms [26] and WEGO was used for GO functional classification of all unigenes [27]. Furthermore, KEGG was used to study the complex biological behavior of the genes and interpret the macroscale distribution of gene functions in the species.

### mRNA quantitative real-time PCR analysis

Total RNA was extracted from the leaf, scion bark, rootstock bark, and root samples of ‘Shatangju’ mandarin grafted onto the different rootstocks (Fragrant orange, Red tangerine, ‘Shatangju’ mandarin, Canton lemon, and Rough lemon) using a Trizol kit (TaKaRa, Japan). DNA contamination was removed using DNase I (TaKaRa, Japan). The quality of the RNA extracts was examined by UV spectrophotometry and agarose gel electrophoresis before being used for single-stranded cDNA synthesis (TaKaRa, Japan).

The quantitative real-time PCR (qPCR) experiments were done according to MIQE précis [28]. Primers were designed using BatchPrimer3, with the Actin gene as an endogenous control gene (S1 Table). qRT-PCR was performed on the LightCycler 480 (Roche, Switzerland) using LightCycler 480 SYBR Green I dye. Amplification efficiencies were determined for each primer pair using a 5-fold dilution series of template plotted into a six-point standard curve. The PCR reaction contained 5 μL SYBR Green I dye, 1 μL cDNA, and 0.4 μL each of the upstream and downstream primers (10 μM) for a final volume of 10 μL. The following PCR procedure was used: an initial denaturation at 95°C for 5 min, 40 cycles of denaturation at 95°C for 15 s, followed by the optimal annealing temperature of each primer (55~63°C) for 1 min, 95°C for 15 s, plus 65°C to 95°C for 15 s (melting curve). Each reaction was done in duplicate, in a 384-well optical-grade PCR plate and sealed with optical sealing tape (Roche, Switzerland). Relative gene expression was calculated using the 2^-ΔΔCT^ method [29]. Data are the means of three biological replicates.

### Statistical analysis

All data were subjected to analysis of variances by using SPSS 18.0 Statistics (SPSS Inc., Chicago, IL, USA). The differences among treatment means were evaluated by Duncan’s multiple range test at a 0.05 probability level. Figures were generated using OriginPro 8.5 (OriginLab Corp., Northampton, MA, USA).

## Results

### Effect of rootstocks on plant growth

**Fig 1:**
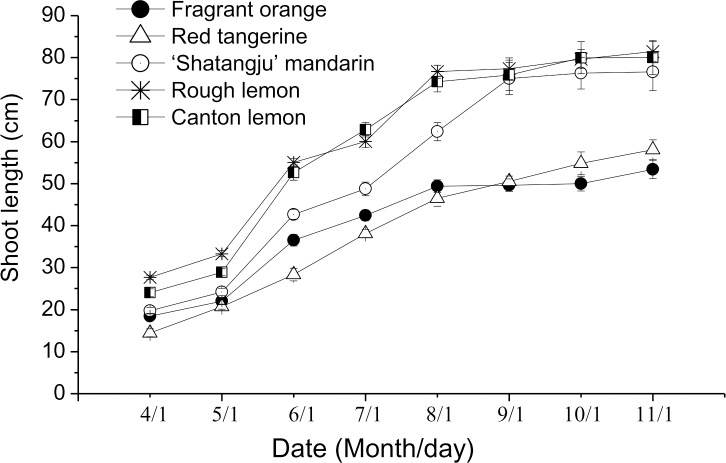
Changes of shoot length in ‘Shatangju’ mandarin grafted onto different rootstocks in 2013. Error bars show the standard error of ten biological replicates.

Rootstocks had a significant effect on the growth vigor of grafted trees (Fig 1). May to August was the fast-growing stage during which shoot growth of the scion showed large differences between the various rootstocks. The most vigorous growth and the longest shoot lengths were obtained for the ‘Shatangju’ mandarin grafted onto Rough lemon and Canton lemon rootstocks. The shortest shoot length and weakest growth vigor of the grafted plants were obtained with Fragrant orange and Red tangerine rootstocks.

Nine months after grafting, significant changes were shown in the growth parameters of the ‘Shatangju’ mandarin grafted onto the different rootstocks (Table 1). The weights of the aboveground part and roots were significantly greater in the ‘Shatangju’ mandarin grafted onto the Rough lemon and Canton lemon rootstocks compared with other combinations. In terms of the trunk diameter of the scion, the values associated to Rough lemon and Canton lemon rootstocks were still the largest, whereas the Fragrant orange and Red tangerine rootstocks were the smallest. Red tangerine and Fragrant orange rootstocks resulted in the smallest single leaf areas. Rough lemon and Canton lemon rootstocks supported longer internodal lengths, whereas the shortest internodal length was obtained on the Red tangerine rootstock. Fragrant orange and Red tangerine rootstocks obtained highest root-shoot ratios.

**Table 1:** Effects of different rootstocks on the growth parameters of ‘Shatangju’ mandarin.

Rootstocks	Weight of overground part(g)	Weight of root(g)	Trunk diameter of scion(mm)	Leaf areas(cm^2^)	Internodal length(cm)	Root-shoot ratio
Fragrant orange	89.65±9.1a	114.25±12.1a	8.8±0.4a	11.13±0.61a	1.9±0.10a	1.27±0.05c
Red tangerine	99.64±5.0a	107.88±8.9a	9.3±0.3a	9.95±0.45a	1.71±0.08a	1.08±0.07b
‘Shatangju’ mandarin	130.45±12.9a	111.65±8.0a	10.9±0.5b	19.02±0.3b	1.84±0.08a	0.85±0.06a
Rough lemon	224.23±28.3b	167.24±27.0b	12.7±0.7c	17.13±0.59b	2.18±0.09b	0.75±0.03a
Canton lemon	247.77±21.8b	170.04±26.3b	14.7±0.6d	19.65±0.7b	2.24±0.05b	0.68±0.06a

The data are means±SE of ten biological replicates. Different letters indicate "significantly different" at P≤0.05 by Duncan’s multiple range tests.

### Hormone levels

**Fig 2:**
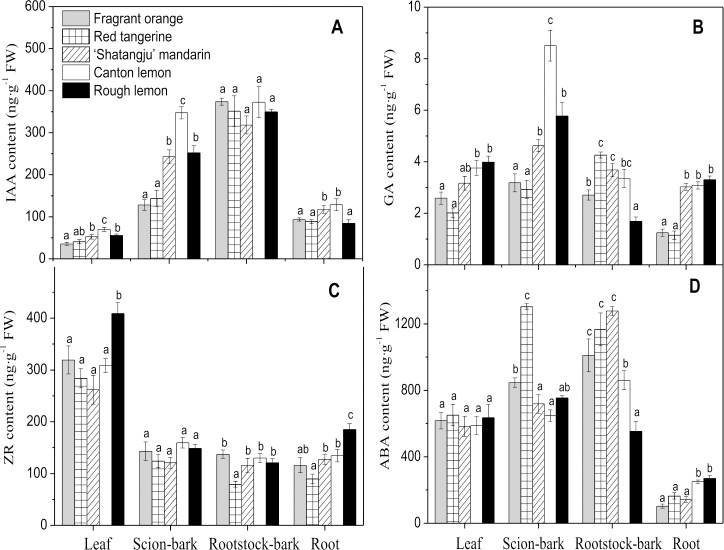
Hormone levels in different tissue of ‘Shatangju’ mandarin grafted onto different rootstocks. (A) indole-3-acetic acid, (B) gibberellin, (C) zeatin riboside, (D) abscisic acid. Error bars show the standard error of five biological replicates. Different letters indicate "significantly different" at P≤0.05 by Duncan’s multiple range tests.

Different rootstocks significantly affected the IAA levels in the grafted ‘Shatangju’ mandarin (Fig 2A). In terms of leaf IAA level, the Canton lemon rootstock was markedly higher than other treatments, whereas leaf IAA levels with Fragrant orange and Red tangerine rootstocks were relatively low. Similar IAA levels were detected in the scion bark, being highest in Canton lemon rootstock and lowest in the Fragrant orange and Red tangerine rootstocks. The IAA levels in the rootstock bark showed no significant differences among the rootstock treatments. Root IAA levels were significantly higher with Canton lemon and self-rooted rootstocks comparatively to other rootstock treatments. Leaf gibberellin (GA) levels were relatively high in the ‘Shatangju’ mandarin grafted onto Rough lemon and Canton lemon rootstocks, whereas low GA levels were obtained with Fragrant orange and Red tangerine rootstocks (Fig 2B). In the scion bark, GA levels were highest with the Canton lemon rootstock, lowest with Fragrant orange and Red tangerine rootstocks. Root GA levels were lowest with Fragrant orange and Red tangerine rootstocks compared with the three other treatments.

Leaf zeatin riboside (ZR) levels were significantly higher with Rough lemon rootstock compared with other treatments (Fig 2C). For the root ZR levels, the result corresponding to Rough lemon rootstock was significantly higher than in the other treatments; ZR levels were the lowest with Red tangerine rootstock. In terms of scion-bark abscisic acid (ABA) levels, result relative to Red tangerine rootstock was relatively high, whereas Canton lemon and Rough lemon rootstocks displayed low values. The opposite trends were observed with root ABA levels; whereas, Canton lemon and Rough lemon rootstocks showed significantly higher values than with the three other treatments.

### mRNA-Seq quality assessment

After sequencing, the number of the original reads was no less than 20 M for each sample, the read length of the sequencing was more than 90 nucleotides (nt), and the percentage of bases with quality value greater than 20 (Q20) was greater than 95%. These results indicate that the quality of the sequencing results was acceptable (Table 2). In total, clean reads accounted for ~95% of the total sequences, low-quality sequences accounted for 0.007%, and sequences only containing the adaptor accounted for ~0.018%. The high proportion of clean reads and low proportion of low-quality or adaptor sequences demonstrate the high quality of the sequencing, which lay the foundation for subsequent information analysis. Using TopHat, the obtained clean reads were compared with a citrus reference genome which was Clementine species (*Citrus reticulata* Blanco). Approximately 85% of the clean reads were mapped onto the reference genome (S2 Table).

**Table 2:** Output statistics of sequencing and mapping.

Sample ID	Q20 Value (%)	Raw reads	Clean reads	Clean ratio	Mapped reads	Mapped Unique reads	Mapping ratio
Red tangerine	95.9	29,238,184	27,745,302	94.9%	23,694,050	22,493,454	85.4%
Canton lemon	96.9	21,427,392	20,350,330	95.0%	17,355,122	16,472,001	85.3%
‘Shatangju’ mandarin	95.7	23,929,248	22,709,554	94.9%	19,214,019	18,285,490	84.6%

Clean ratio = (Clean reads/Raw reads) ×100%

### GO term enrichment analysis

**Fig 3:**
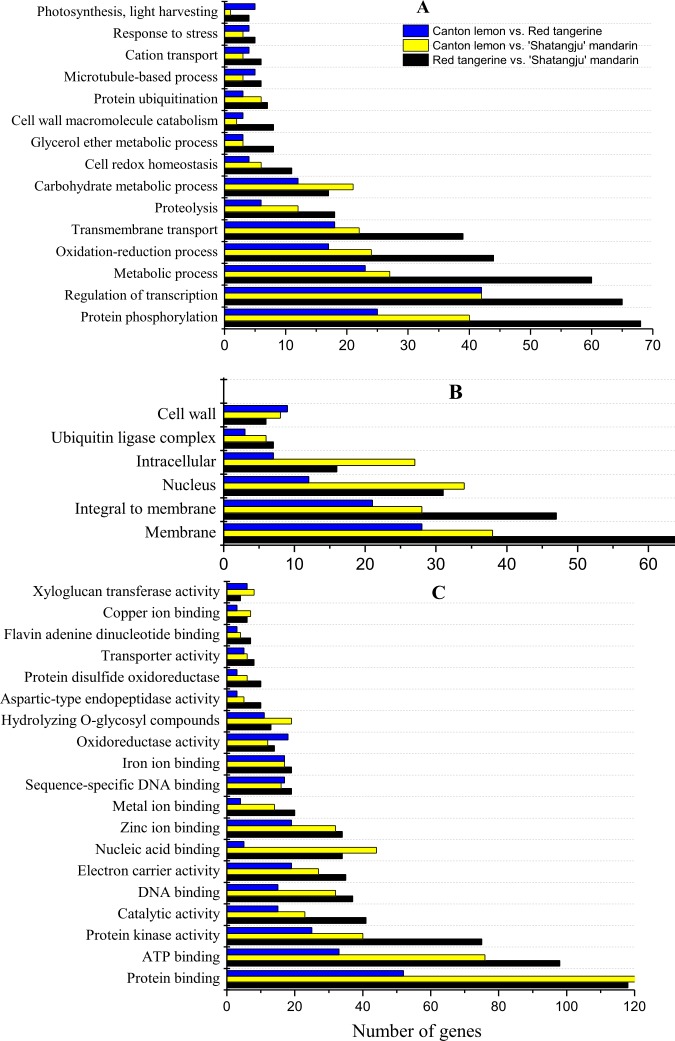
Gene ontology analysis of differentially expressed genes of Canton lemon vs. Red tangerine, Canton lemon vs. ‘Shatangju’ mandarin, and Red tangerine vs. ‘Shatangju’ mandarin. A, biological process; B, cellular component; C, molecular function.

Significance analysis of the GO function was performed for all differentially expressed genes (DEGs) of Red tangerine vs. ‘Shatangju’ mandarin, Canton lemon vs. ‘Shatangju’ mandarin, and Canton lemon vs. Red tangerine (Fig 3, S3 Table). The most DEGs with GO annotations were obtained for Red tangerine vs. ‘Shatangju’ mandarin, whereas the fewest genes were obtained for Canton lemon vs. Red tangerine. However, more DEGs were enriched in the cell wall and showed oxidoreductase activity for Canton lemon vs. Red tangerine. Regarding the biological processes, GO terms enriched with DEGs included protein phosphorylation, transcriptional regulation, metabolic process, redox processes, and transmembrane transport. In the cellular components, the membrane component was relatively enriched with DEGs. Molecular functions involved the most DEGs, and the GO terms enriched with DEGs included protein, ATP, and DNA binding; protein kinase and catalyzing enzyme activity; electron carrier and metal ion binding.

### KEGG analysis

**Fig 4:**
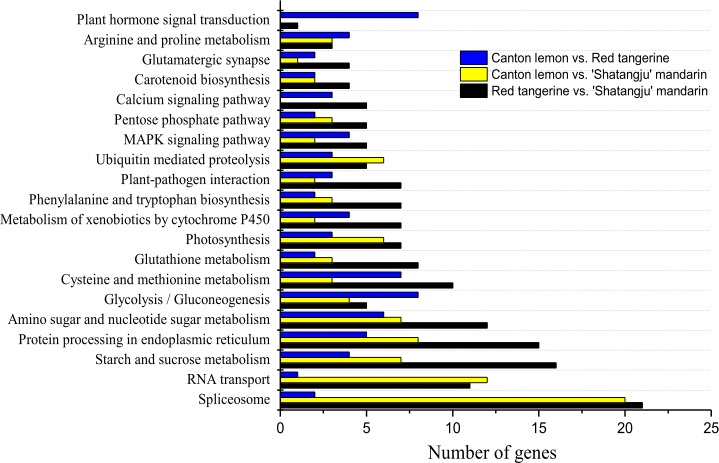
KEGG pathway analysis of differentially expressed genes of Canton lemon vs. Red tangerine, Canton lemon vs. ‘Shatangju’ mandarin, and Red tangerine vs. ‘Shatangju’ mandarin.

The KEGG pathway annotations showed that 546 DEGs were annotated to 165 metabolic pathways for Red tangerine vs. ‘Shatangju’ mandarin; 366 DEGs were annotated to 136 metabolic pathways for Canton lemon vs. ‘Shatangju’ mandarin; and 258 DEGs were annotated to 121 metabolic pathways for Canton lemon vs. Red tangerine. Fig 4 illustrates a portion of the significantly enriched (P-value ≤ 0.05) metabolic pathways. The most DEGs were enriched in spliceosome, RNA transport, and starch and sucrose metabolism. Meanwhile, the DEGs were significantly enriched in the plant hormone signal transduction pathway and glycolysis/gluconeogenesis for Canton lemon vs. Red tangerine, whereas fewer DEGs were enriched compared with the other two groups (S4 Table).

### Cluster analysis

**Fig 5:**
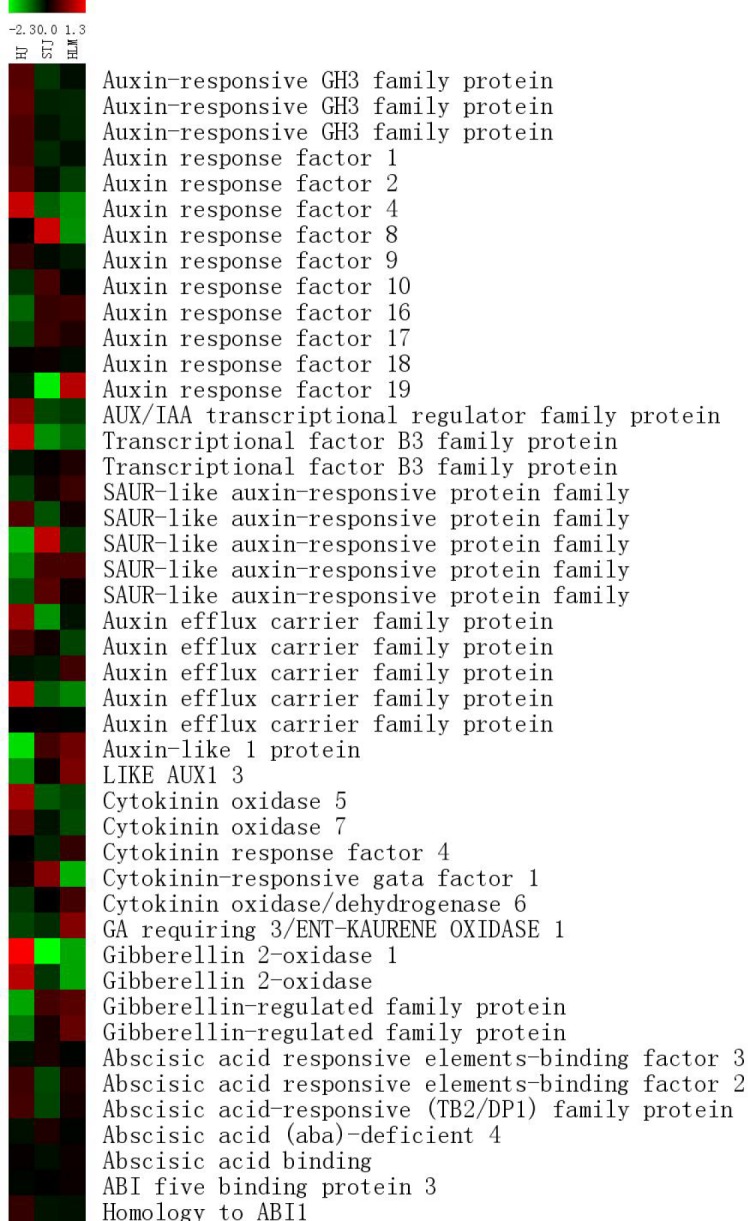
A comparative heat map shown for genes related to hormone signal transduction from mRNA-Seq data. HJ, STJ and HLM are used to refer specifically to the ‘Shatangju’ mandarin grafted onto the Red tangerine, ‘Shatangju’ mandarin and Canton lemon rootstocks, respectively.

Fig 5 shows the gene-expression profiles related to the signal transduction pathway of four hormones. In total, we screened out 29 genes related to auxin signal transduction, ~60% of which were upregulated in the leaves of ‘Shatangju’ mandarin on the Red tangerine rootstock. The genes included auxin efflux carrier (*PIN*), *GH3*, and most auxin response factors (*ARF*). Some of the *ARF* and *SAUR* genes were upregulated in the leaves of ‘Shatangju’ mandarin grafted onto the Canton lemon rootstock. Among the five GA-related genes, three genes related to the GA signal transduction were upregulated, whereas two genes encoding GA 2-oxidase were suppressed in the leaves of ‘Shatangju’ mandarin on the Canton lemon rootstock. Additionally, cytokinin oxidase-encoding genes were downregulated in leaves of ‘Shatangju’ mandarin on the Canton lemon rootstock. Most genes involved in the ABA signal transduction pathway were downregulated in the leaves of ‘Shatangju’ mandarin on the self-rooted rootstock.

**Fig 6:**
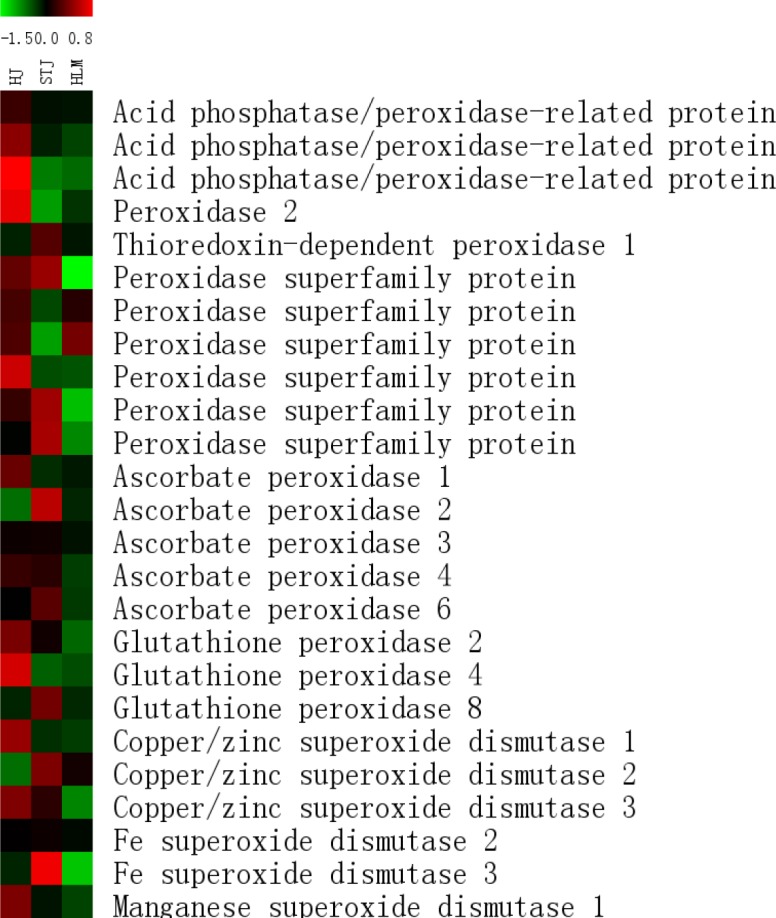
A comparative heat map shown for genes related to antioxidant enzymes from mRNA-Seq data. HJ, STJ and HLM are used to refer specifically to the ‘Shatangju’ mandarin grafted onto the Red tangerine, ‘Shatangju’ mandarin and Canton lemon rootstocks, respectively.

Among the 25 antioxidant enzyme genes, more than 60% were upregulated in the leaves of ‘Shatangju’ mandarin on the Red tangerine rootstock (Fig 6). The upregulated genes included five for peroxidase, three for acid phosphatase, and three for superoxide dismutase. Nine antioxidant enzyme genes were upregulated in the leaves of ‘Shatangju’ mandarin on the self-rooted rootstock, whereas the expression of the antioxidant enzyme genes was almost suppressed in the leaves of ‘Shatangju’ mandarin on the Canton lemon rootstock. The results indicate that the different rootstocks strongly affected the expression of the antioxidant enzyme genes in the scion.

### qRT-PCR analysis of genes in rootstock–scion interaction

**Fig 7:**
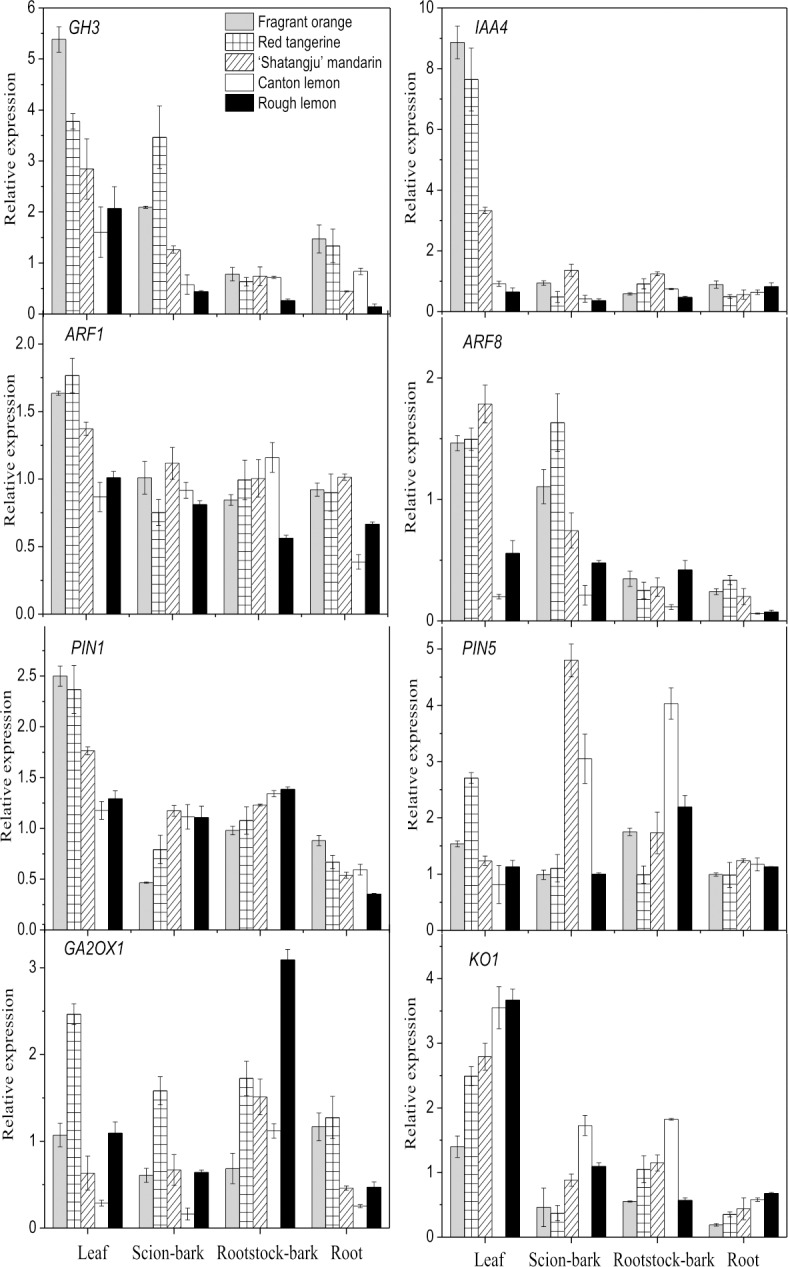
qRT-PCR analysis of genes related to IAA and GA signaling in ‘Shatangju’ mandarin. Data indicating relative transcript level from qRT-PCR are means of three biological replicates.

We screened out 14 genes that play key roles in the regulation of scion growth by rootstocks, and their expression levels were measured by qRT-PCR analysis. The gene expression levels showed consistent trends as those observed in the sequencing data, indicating that the results of the transcriptome sequencing were reliable in this study. *GH3* gene expression in the leaves, scion bark, and roots of ‘Shatangju’ mandarin was higher in the Fragrant orange and Red tangerine rootstocks and lower in the Canton lemon and Rough lemon rootstocks (Fig 7). Leaf expression of the *IAA4* gene was negatively correlated with the growth vigor of the grafted plants, and no clear trends were observed in other parts of the plants. The *ARF1* gene was expressed at low levels in the leaves and roots of the ‘Shatangju’ mandarin on the Canton lemon and Rough lemon rootstocks, whereas the *ARF8* gene was downregulated in various parts of the ‘Shatangju’ mandarin on the Canton lemon rootstock. The leaf and root expression of the *PIN1* gene was downregulated with increasing growth vigor of the grafted plants; the opposite trend was observed in scion bark and rootstock bark. *PIN5* gene expression in the bark was highest on the Canton lemon rootstock. *GA2OX1* gene expression showed similar trends in the leaves, scion bark, and roots of the grafted plants, being the highest on the Red tangerine and lowest on the Canton lemon rootstock. Leaf and root expression of the *KO1* gene was upregulated with increasing growth vigor of the grafted plants, whereas its expression in scion bark and rootstock bark was highest on the Canton lemon rootstock and relatively low on the Fragrant orange and Red tangerine rootstocks.

**Fig 8:**
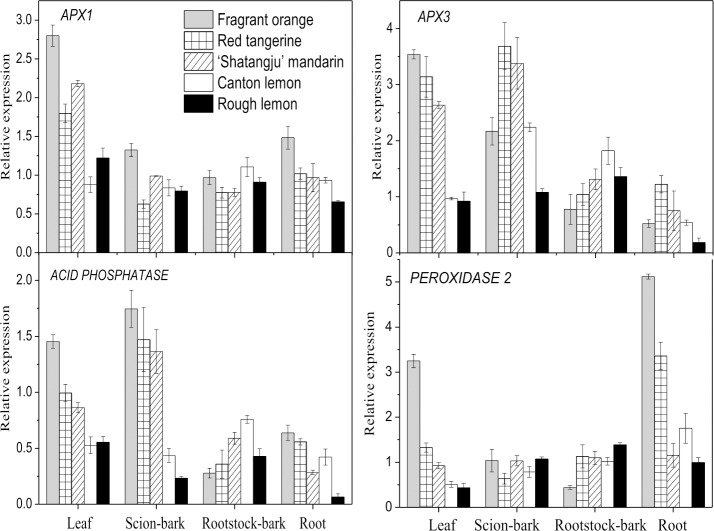
qRT-PCR analysis of genes related to antioxidant enzymes in ‘Shatangju’ mandarin. Data indicating relative transcript level from qRT-PCR are means of three biological replicates.

**Fig 9:**
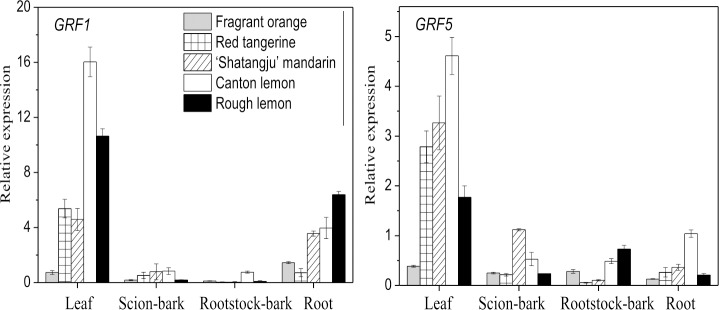
qRT-PCR analysis of genes related to growth regulating factors in ‘Shatangju’ mandarin. Data indicating relative transcript level from qRT-PCR are means of three biological replicates.

Four selected antioxidant enzyme genes showed high expression in the leaves and roots of the ‘Shatangju’ mandarin on the Red tangerine and Fragrant orange rootstocks, whereas their expression was low in the same parts of the ‘Shatangju’ mandarin grafted on the Canton lemon and Rough lemon rootstocks (Fig 8). Likewise, acid phosphatase (*ACPP*) and *APX3* gene expression in the scion bark was negatively correlated with the growth vigor of the grafted plants; however, a positive correlation was observed in the rootstock bark. No clear trends were observed in the *APX1* and *PEROXIDASE 2* gene expression levels in the scion bark or the rootstock bark. The *GRF1* gene was highly expressed in the leaves and roots of the grafted plants, whereas substantially lower expression was detected in the bark (Fig 9). Leaf expression of the *GRF1* gene was highest on the Canton lemon rootstock, followed by the Rough lemon rootstock; the lowest expression was detected on the Fragrant orange rootstock. Root expression of the *GRF1* gene was highest on the Rough lemon rootstock and lowest on the Fragrant orange and Red tangerine rootstocks. For the *GRF5* gene, expression in the leaves was highest with the Canton lemon rootstock and lowest with the Fragrant orange rootstock; similarly, its expression in the scion bark, rootstock bark, and roots was highest on the Canton lemon rootstock and was relatively lower with the Fragrant orange and Red tangerine rootstock.

## Discussion

Rootstocks have a significant effect on the various growth traits of the grafted cultivars of fruit trees. Santos et al. [30] compared the growth traits of sweet cherry grafted onto different rootstocks and found that rootstocks strongly affected the trunk cross-sectional area, shoot length, and internode length of sweet cherry. Similar results have been reported in peach trees. For example, the trunk diameter of peach trees showed a 2.5-fold variation range on the different rootstocks [31]. There were marked differences in the tree height and crown size of the Clementine grafted onto the different rootstocks; both parameters were 10% smaller on dwarf rootstock than on vigorous rootstock [32]. Gijon et al. [33] found that the leaf area and leaf dry weight of pistachio were markedly different on different rootstocks. For grape, the weight of the pruned branches as a growth indicator is also regulated by the rootstock [34]. In the present study, the grafted plants showed the most vigorous growth on the Rough lemon and Canton lemon rootstocks. Nine months after grafting, the weights of both the aboveground part and the roots were markedly higher on the above two rootstocks compared with other rootstock–scion combinations; the trunk diameter of the scion and internodal length were also greatest on the above two rootstocks, showing vigorous characteristics (Table 1, Fig 1). In contrast, the ‘Shatangju’ mandarin grafted onto Fragrant orange and Red tangerine rootstocks had the shortest shoot length, smallest trunk diameter of the scion, shortest internodal length, and the weakest growth vigor, showing dwarfing characteristics.

### Role of endogenous hormones in rootstock–scion interaction

IAA is subject to polar transport from the top to the bottom [35, 36]. Numerous studies suggest that IAA, an information transmitter between the rootstock and the scion, plays a major role in the regulation of scion growth by rootstocks [37, 38]. The strong evidence is that polar IAA transport through the phloem is blocked in plants grafted with an inverted bark graft or grafted onto a dwarf interstock, thereby resulting in dwarf trees [9, 39]. In the present study, the leaves and scion bark IAA levels were positively correlated with the growth vigor of the grafted plants; both were highest on the Canton lemon rootstock and relatively low on the Fragrant orange and Red tangerine rootstocks (Fig 2A). Similarly, Noda et al. [40] noted that shoot IAA levels were highest in the lemon grafted onto a vigorous rootstock ‘Swingle’ citrumelo (*P*. *trifoliata* x *Citrus paradisi*), whereas the lowest IAA levels were detected with dwarf trifoliate orange rootstock. Additionally, root IAA levels were highest with the Canton lemon rootstock, possibly because of high IAA transport downward from the scion. Isotope labeling experiments demonstrated that rootstocks affected IAA transport toward the roots; the amount of IAA received by the roots of apple grafted on the dwarf rootstock was markedly reduced compared with that obtained on vigorous rootstock [41]. The above findings indicated that different rootstocks significantly affected the distribution of the IAA and its transport to the roots of the grafted plants, thus resulting in different growth vigor of the scions.

Gibberellin can promote stem and leaf elongation. The effect of the GA, which is based on the IAA change, has a synergistic effect with IAA. Meanwhile, GA can affect the IAA activity [42, 43]. Therefore, GA has been proposed as the most important hormone for controlling tree size [44]. In the present study, leaf, scion bark, and root GA levels of the grafted plants were highest on the Canton lemon and lowest on the Red tangerine rootstock; a positive correlation was observed between the GA level and the plant growth vigor (Fig 2B). GA can be synthesized in the roots and transported by sap to the shoots through transpiration, thereby influencing tree growth [45]. Van-Hooijdonk et al. [46] found that GA19 (precursor of GA1 synthesis) levels were lower in the xylem of the apple grafted onto the dwarf rootstock M.9. They suggested that the dwarf rootstock of the apple limited the transport of the GA19 produced by the roots to the shoots and thus resulted in dwarf trees. Meanwhile, we found a positive correlation between the GA and IAA levels in the leaves, scion bark, and roots of the grafted plants. Auxin can facilitate the biosynthesis and signal transduction of GA. This action by auxin is a necessary condition for plant synthesis of active GA [43, 47]. Exogenous auxin transport inhibitor treatment has been shown to markedly reduce GA1 levels in the shoots of pea and tobacco [48, 49]. Therefore, dwarf rootstock can be hypothesized to limit IAA transport in the scion from the top down and to reduce the amount of IAA received by the roots. This mechanism limits GA synthesis in the roots and its transport to the aboveground part, thereby reducing GA levels in the scion and resulting in dwarf trees [46, 50].

### Transcriptome sequencing and DEG screening of ‘Shatangju’ mandarin leaves on different rootstocks

In previous studies, cDNA-AFLP and gene chip technology have been used to investigate transcriptome changes in the different rootstock–scion combinations of fruit tress including sweet cherry [7], apple [8], and grape [11]. A number of DEGs associated with the growth vigor of plants have been identified, such as brassinosteroid response genes, leucine zipper protein genes, and expansin genes. In the present study, we used RNA-Seq to examine transcriptome differences in the leaves of ‘Shatangju’ mandarin grafted onto different rootstocks and explored genes playing a critical role in the rootstock-scion interaction. DEGs were identified among the three leaf samples of ‘Shatangju’ mandarin grafted onto Red tangerine, ‘Shatangju’ mandarin, and Canton lemon rootstocks. More than 1000 DEGs were found, and these involve more than 100 functional categories and metabolic pathways. Moreover, we found more DEGs enriched in the oxidoreductase function, hormone signal transduction, and glycolytic pathways in ‘Shatangju’ mandarin grafted onto the Canton lemon vs. Red tangerine that showed large difference in growth vigor (Figs 4 and 5). This implied that genes relate to the above functions and pathways played a pivotal role in the effect of the rootstock on the scion growth.

### Role of genes related to hormonal signaling in rootstock–scion interaction

Auxin can induce rapid and transient expression of certain genes, the so-called primary auxin-responsive genes. There are three major classes of auxin-responsive genes: the Aux/IAA, SAUR, and GH3 gene families [51]. GH3 proteins have synthase activity for auxin–amino acid conjugates and catalyze free-state IAA to bind amino acids, thereby deactivating IAA to reduce the auxin signal [52]. The Aux/IAA gene family comprises important transcriptional repressors in the auxin signal transduction pathways. Aux/IAA transcription factors can respond to auxin and rapidly degrade, thereby initiating the auxin signal transduction pathway [53, 54]. In the present study, *GH3* gene expression in leaves, scion bark, and roots was negatively correlated with the growth vigor and IAA levels of grafted ‘Shatangju’ mandarin. Similarly, leaf expression of the Aux/IAA family gene *IAA4* was downregulated with increasing growth vigor and IAA levels in the grafted ‘Shatangju’ mandarin. The above results indicate that the selective expression of genes from the GH3 and Aux/IAA families played a critical role in the auxin signaling-mediated growth regulation of the grafted plants. Kloosterman et al. [55] also showed that the downregulation of *IAA2* expression led to increased plant height and reduced bending of apical leaf primordia in the potato.

Auxin response factors are transcription factors that mediate auxin reactions and regulate auxin response gene expression [56, 57]. In the present study, leaf and root expression of the *ARF1* gene was substantially lower in ‘Shatangju’ mandarin grafted onto the Canton lemon and Rough lemon rootstocks. Additionally, *ARF8* gene expression was suppressed in various parts of ‘Shatangju’ mandarin grafted onto the Canton lemon rootstock. However, the expression of both the *ARF1* and *ARF8* were upregulated in ‘Shatangju’ mandarin grafted onto the Fragrant orange and Red tangerine rootstocks with low growth vigor (Fig 7). ARF proteins can be divided into transcriptional activators and repressors. ARF1, as a transcriptional repressor, plays a role in inhibiting the transcription of auxin response genes [58] and negatively regulating plant growth vigor and organ size [56]. Likewise, ARF8 plays a negative regulatory role in plant growth and development [59, 60]. For instance, ARF8-overexpressing transgenic Arabidopsis showed shorter hypocotyls, weaker apical dominance, and lower free-state IAA levels [61]. As has been shown, the ARF8 transcription factor activates the GH3 family genes for transcriptional regulation [62]. This is in agreement with the results of the present qRT-PCR analysis. Therefore, we conclude that the upregulation of *ARF8* gene expression stimulated *GH3* transcription in ‘Shatangju’ mandarin grafted onto the Fragrant orange and Red tangerine rootstocks. This mechanism deactivated IAA and attenuated the effect of auxin, leading to reduced growth vigor in the grafted plants.

The transmembrane transport of auxin between plant cells depends on the carrier proteins in the cell membrane: the auxin influx carriers belong to the AUX/LAX protein family, and the auxin efflux carriers belong to the PIN protein family [63]. The current results showed that leaf *PIN* expression was highest in ‘Shatangju’ mandarin grafted onto the Red tangerine rootstock; the lowest levels were detected on the Canton lemon and Rough lemon rootstocks with high growth vigor. This might reduce the efflux of auxin from the leaves, thereby facilitating the accumulation of auxin and maintaining vigorous growth in this part. The opposite trend was observed in the scion bark and rootstock bark; that is, *PIN* expression was the highest on the Canton lemon and lowest on the Red tangerine and Fragrant orange rootstocks (Fig 7). Low *PIN* expression in the bark of ‘Shatangju’ mandarin grafted onto the Red tangerine rootstock could suppress the IAA transport to the roots and thus reduce the amount of IAA received by these latter. Consequently, root growth and relevant hormone synthesis were restricted, thereby affecting shoot growth. Li et al. [9] have noted that the M9 interstock results in dwarf apple because the low *PIN* gene expression in the phloem of the interstock limits the IAA transport downward and leads to IAA deficiency in the roots. This conclusion is similar to our results. Additionally, the application of NPA (auxin transport inhibitor) to the shoots of the vigorous rootstock could markedly inhibit the shoot growth of the scion [46].

Ent-kaurene oxidase is a key enzyme in the second stage of the GA biosynthesis. This enzyme catalyzes kaurene to produce GA12-aldehyde, the initial product of GA, through a series of hydroxylation reactions [64]. When this function is lacking or the synthesis of ent-kaurene oxidase is blocked, the shoot and internodal lengths are shortened, and root growth is prevented [64, 65]. The role of GA2ox in the GA biosynthesis and metabolic pathways is to convert active GA and its precursors into irreversible inactive forms of GA [66]. In the present study, *KO1* gene expression was positively correlated with the growth vigor and GA levels of grafted ‘Shatangju’ mandarin; in contrast, *GA2OX1* gene expression was negatively correlated with plant growth vigor (Fig 7). The GA2OX1 gene uses C19-GA as a substrate to convert bioactive GA1 and GA4 into inactive GA8 and GA34 [67]. In GA2OX1-overexpressing Arabidopsis [68], wheat [69], potato [70], and rice [71], all transgenic plants exhibited a dwarf phenotype and markedly reduced the bioactive GA levels. In short, high *GA2OX1* expression and low *KO1* gene expression resulted in decreased levels of total and active GA in the ‘Shatangju’ mandarin grafted onto the Red tangerine rootstock, thereby reducing plant growth vigor. Meanwhile, the *GA2OX1* gene was negatively regulated by auxin and thus affected the GA metabolism [72].

### Expression of antioxidant enzymes and growth-regulating factors

Our transcriptome data showed that most antioxidant enzyme genes were upregulated in the leaves of ‘Shatangju’ mandarin on the Red tangerine rootstock; however, almost all the antioxidant enzyme genes were suppressed in the leaves of ‘Shatangju’ mandarin grafted on the Canton lemon rootstock (Fig 6). As revealed by the qRT-PCR analysis, both leaf and root expression of the four selected antioxidant enzyme genes were negatively correlated with the growth vigor of the grafted plants (Fig 8). Meanwhile, the gene expression showed a positive correlation with antioxidant enzyme activity in the grafted plants. Peroxidases are involved in the different physiological functions in the plants, such as peroxide scavenging, lignification, cell wall synthesis, and auxin metabolism [73, 74]. Transgenic experiments showed that both cell elongation and hypocotyl length were suppressed in *AtPRX53* (a *PEROXIDASE* 2 homologous gene)-overexpressing transgenic plants, whereas hypocotyl length was increased in an AtPRX53 mutant [75]. Further analysis confirmed that the effect of *AtPRX53* overexpression on cell elongation was associated with a cross-linking reaction in the cell walls. Cosio et al. [76] showed that overexpression of the anionic peroxidase gene *APRX* significantly reduced endogenous IAA levels and ceased the growth of transgenic plants. Meanwhile, APRX exhibited auxin oxidase activity in *in vitro* experiments, which confirmed the role of peroxidase in auxin metabolism. Furthermore, exogenous auxin can inhibit the activity and gene expression of *APX1* [77]. Therefore, significant antioxidant enzyme gene expression could be induced to enhance peroxidase activity in grafted plants with low growth vigor. This might accelerate auxin metabolism and the cross-linking of the structural proteins, hemicellulose, and pectin in the cell wall, ultimately hindering cell elongation.

Growth-regulating factors (GRFs) are an important class of transcription factor proteins in plants. GRFs commonly play a positive regulatory role in plant growth and development [78, 79]. Studies on *Arabidopsis* and rice showed that *GRF* genes were highly expressed in young tissues including growing roots, leaves, and flowers; the gene expression was substantially lower or was not observed in mature organs or tissues [80, 81]. Similarly, our results showed that the *GRF* gene was highly expressed in the leaves and roots of the grafted plants, whereas its expression was significantly lower in the bark. Additionally, *GRF1* and *GRF5* gene expression was relatively higher in the ‘Shatangju’ mandarin grafted onto Canton lemon and Rough lemon rootstocks associated with vigorous growth, whereas their expression was lowest in the Fragrant orange rootstock (Fig 9). Luo et al. [78] used RNA interference to reduce *GRF1* gene expression in rice, which resulted in smaller leaf area, shorter plant height, and delayed heading stage. Transgenic experiments showed that *GRF1* and *GRF5* overexpression in *Arabidopsis* facilitated the growth of plant leaves and cotyledons; the leaf size change was mainly due to cell number change in transgenic plants [79, 80, 82]. Therefore, the differential expression of the GRF transcription factors affected cell proliferation in the growing tissues and thus regulated the plant growth of ‘Shatangju’ mandarin grafted onto the different rootstocks.

In summary, our results have demonstrated that citrus rootstocks exert a significant effect on the plant growth of grafted trees. Endogenous hormones such as IAA and GA play an important role in the interaction of rootstock and scion. Citrus rootstock affected the synthesis, transportation and distribution of endogenous hormones in the grafted plants, leading to the different growth potential. RNA-Seq data showed that genes involved in the oxidoreductase function, hormone signal transduction, and glycolytic pathways may play a pivotal role in the effect of the rootstock on the scion growth. Different rootstocks significantly affected the expression of genes involved in auxin signal transduction pathway and GA biosynthesis pathway in the grafted plants, and then regulated the hormone levels and their signal pathways. The differential expression of these genes may be involved in the growth regulation by citrus rootstocks. Moreover, our present results indicate that antioxidant enzymes and growth-regulating factors may be involved in grafted plant growth regulation. Multiple pathways likely exist and independently regulate the plant growth in the process of the rootstock–scion interaction.

## Supporting Information

S1 Table Primers used for real-time quantitative RT-PCR. (DOC)

S2 Table Differentially expressed unigenes between the rootstock treatments. (XLS)

S3 Table Enrichment GO anotation of the differential expression genes between the rootstock treatments. (XLS)

S4 Table KEGG pathway annotation of the differential expression genes between the rootstock treatments. (XLS)
