# Supplementary material for: Transcriptome Profiling to Understand the Effect of Citrus Rootstocks on the Growth of ‘Shatangju’ Mandarin
Source: PLoS One. 2017 Jan 12;12(1):e0169897. doi: 10.1371/journal.pone.0169897 (PMC5231354; doi:10.1371/journal.pone.0169897)
Supplement: S1 Table — (DOC) [file pone.0169897.s001.doc]

**Supplementary Table S1. Primers used for real-time quantitative RT-PCR.**

| Gene name | Description | Primer sequence （5' to 3'） |
| --- | --- | --- |
| *GH3* | GH3 family protein | AGTTATGGCTGCGTCTGCTT |
|  |  | GGCAGTACATTTGGTGCTGA |
| *IAA4* | AUX/IAA family protein | TGGGGCGCCATATCTAAGGA |
|  |  | AGCATCCAGTCGCCATCTTT |
| *PIN1* | Auxin efflux carrier protein 1 | GTTGATGGACCTGGAAGTGC |
|  |  | GCTAATGTGACGATGCCAAG |
| *PIN5* | Auxin efflux carrier protein 5 | CGTCAACACTAAAGCCACTGA |
|  |  | GGTCTGATGGAAGGAAACCA |
| *ARF1* | Auxin response factor 1 | GGGCTGTTGATTTGACACG |
|  |  | CCACTTGCCACTTCTTGGTT |
| *ARF8* | Auxin response factor 8 | CTCCCAACAAACAGCCAAC |
|  |  | ATCCCTCGCAATCAGTTCTT |
| *GA2OX1* | GA2-oxidase1 | CTGGCCTCCAAATCCAACT |
|  |  | GTCATTACCTGCAAGGCATC |
| *KO1* | Ent- kaurene oxidase | CAAGACTGGCGCTTCTACTA |
|  |  | TGGGTGGAAATGCTCAGAAGC |
| *ACID PHOSPHATASE* | Acid phosphatase | TTCAGTTGGGCTGTTGTTTG |
|  |  | CCTTTGCGACGATAGAGCAC |
| *APX1* | Ascorbate peroxidase 1 | TTCCCTACCATCTCCTACGC |
|  |  | CTCAGCCTTGTCATCTCTTCC |
| *APX3* | Ascorbate peroxidase 3 | AGGAAATCGAGAAGGCTCGT |
|  |  | TCGCATCATAAGTTCCAGCA |
| *PEROXIDASE 2* | Peroxidase | AGCAGAACAGCCAACAGGAC |
|  |  | CACTGAGCCCTTCCAAATGT |
| *GRF1* | Growth regulating factor 1 | CTTGGTTATTCGGGCAACAC |
|  |  | CCTTCCACAGGCTTTCTTGA |
| *GRF5* | Growth regulating factor 5 | CGAAATCCAGCAACACCTC |
|  |  | TCGGAGAGACTGACCTTCCT |
| *Actin* | Actin protein | CCAAGCAGCATGAAGATCAA |
|  |  | ATCTGCTGGAAGGTGCTGAG |
